# A computer aided measurement method for unstable pelvic fractures based on standardized radiographs

**DOI:** 10.1186/s12880-015-0084-x

**Published:** 2015-09-30

**Authors:** Jing-xin Zhao, Zhe Zhao, Li-cheng Zhang, Xiu-yun Su, Hai-long Du, Li-ning Zhang, Li-hai Zhang, Pei-fu Tang

**Affiliations:** Department of Orthopedics, Chinese PLA 82nd Hospital, No.100 East Jiankang Road, Qinghe District, Huai’an, Jiangsu Province 223001 People’s Republic of China; Department of Orthopedics, Chinese PLA General Hospital, No.28 Fuxing Road, Beijing, Haidian District 100853 People’s Republic of China; Department of Orthopedics, Beijing Tsinghua Chang Gung Hospital, No.1 Block Tiantongyuan North, Beijing, 102218 People’s Republic of China; Department of Orthopedics, Affiliated Hospital of the Academy of Military Medical Sciences, No.8 Dongdajie Road, Beijing, 100071 People’s Republic of China

**Keywords:** Unstable pelvic fractures, Radiographic assessment, Software-based measurement, Reliability study

## Abstract

**Background:**

To set up a method for measuring radiographic displacement of unstable pelvic ring fractures based on standardized X-ray images and then test its reliability and validity using a software-based measurement technique.

**Methods:**

Twenty-five patients that were diagnosed as AO/OTA type B or C pelvic fractures with unilateral pelvis fractured and dislocated were eligible for inclusion by a review of medical records in our clinical centre. Based on the input pelvic preoperative CT data, the standardized X-ray images, including inlet, outlet, and anterior-posterior (AP) radiographs, were simulated using Armira software (Visage Imaging GmbH, Berlin, Germany). After representative anatomic landmarks were marked on the standardized X-ray images, the 2-dimensional (2D) coordinates of these points could be revealed in Digimizer software (Model: Mitutoyo Corp., Tokyo, Japan). Subsequently, we developed a formula that indicated the translational and rotational displacement patterns of the injured hemipelvis. Five separate observers calculated the displacement outcomes using the established formula and determined the rotational patterns using a 3D-CT model based on their overall impression. We performed 3D reconstruction of all the fractured pelvises using Mimics (Materialise, Haasrode, Belgium) and determined the translational and rotational displacement using 3-matic suite. The interobserver reliability of the new method was assessed by comparing the continuous measure and categorical outcomes using intraclass correlation coefficient (ICC) and kappa statistic, respectively.

**Result:**

The interobserver reliability of the new method for translational and rotational measurement was high, with both ICCs above 0.9. Rotational outcome assessed by the new method was the same as that concluded by 3-matic software. The agreement for rotational outcome among orthopaedic surgeons based on overall impression was poor (kappa statistic, 0.250 to 0.426). Compared with the 3D reconstruction outcome, the interobserver reliability of the formula method for translational and rotational measures was perfect with both ICCs more than 0.9.

**Conclusions:**

The new method for measuring displacement using a formula was reliable, and could minimise the measurement errors and maximise the precision of pelvic fracture description. Furthermore, this study was useful for standardising the operative plan and establishing a theoretical basis for robot-assisted pelvic fracture surgery based on 2-D radiographs.

## Background

The most severe injury observed in an orthopaedic trauma centre are disruptions of the pelvic ring, especially unstable pelvic ring fractures, which are characterized as a posterior pelvic ring fracture with the partial or total displacement of unilateral or bilateral pelvis. In cases of increased displacement and the incidence of complications from fractured pelvis, it is much more difficult to manage these types of complicated fractures. Traditionally, the fracture displacement is assessed using radiological tools, including roentgenography and CT scans. As radiographic assessment remains the standard for preoperative assessment, the first step of management is rapid and precise measurement and the determination of the displacement of the injured pelvis. For a long time, CT based measurements method of pelvic displacements was considered as the “gold standard” [1, 2]. However, regarding the higher cost and patients’ radiation exposure of CT scan, the imaging examinations based on the radiographs were still the most available and convenient tools in most clinical settings.

A review of the literature revealed that displacement assessments of the pelvis relied on three-direction roentgenography, including inlet, outlet and anterior-posterior (AP) plain radiographs [3–9]. As with other outcome measurement tools, reliability and validity of radiographic measurement methods are of utmost importance if there is an intention or attempt to use them to conduct pre- or post-operative assessment and to correlate them with an operation’s outcome. However, the reliability and validity of current measurement techniques are not strong [8, 10], because of patient factors (the presence of overlapping osseous structures, bowel gas and intestinal contrast materials) and technical factors (unstandardized technique, deficiency of validation) [2].

Nystrom et al. used the Sawbones model to simulate unstable pelvic ring fracture patterns and reconstructed the 3-dimensional (3D) model based on CT scan data [11]. After measuring the pelvic displacement using 3 previous established methods, they found that the measurement of vertical translation and sacroiliac (SI) joint separation as described by Henderson et al. was reliable [3], and the measurement of SI joint displacement as described by Matta et al. was difficult to make and unreliable [7]. Previous measurement techniques mainly focused on translational displacement of the pelvis, whereas unstable pelvic ring fractures often lead to translational or rotational displacement of the pelvis in different planes in 3D space. Solely measuring translational displacement without considering rotational displacements not only increased the measurement error but also neglected important anatomic information, which underlied the following treatment plan.

We simulated standardized X-ray images using Armira software and hypothesized that some formulas can be developed to precisely measure and indicate the translational and rotational displacements of the injured hemipelvis in each plane based on the 2D coordinate information of those representative anatomic landmarks. Ideally, this study could be used to standardize the operative plan and establish a theoretical basis for robot-assisted surgery based on 2D radiographs.

## Methods

### Design and setting

The study was a medical imaging investigation approved by the Ethics Committee of Chinese PLA General Hospital. Due to its retrospective nature and the anonymous patient data, a waiver of patients’ informed consent was granted. Data were obtained on patients with pelvic fractures who were admitted to the Department of Orthopaedics and Trauma at the Chinese PLA General Hospital between March 2012 and March 2013.

A review of plain radiographs revealed that 25 patients with unilateral pelvis injury and dislocation had minimal or no displacement of the contralateral segment. We collected the CT Digital-Imaging-and-Communications-in-Medicine (DICOM) data. All CT scans were performed with a Somatom sensation open CT System (Siemens AG, Erlangen, Germany) with slice thicknesses of 1.5 mm.

### Pelvis specific coordination system

**Fig. 1:**
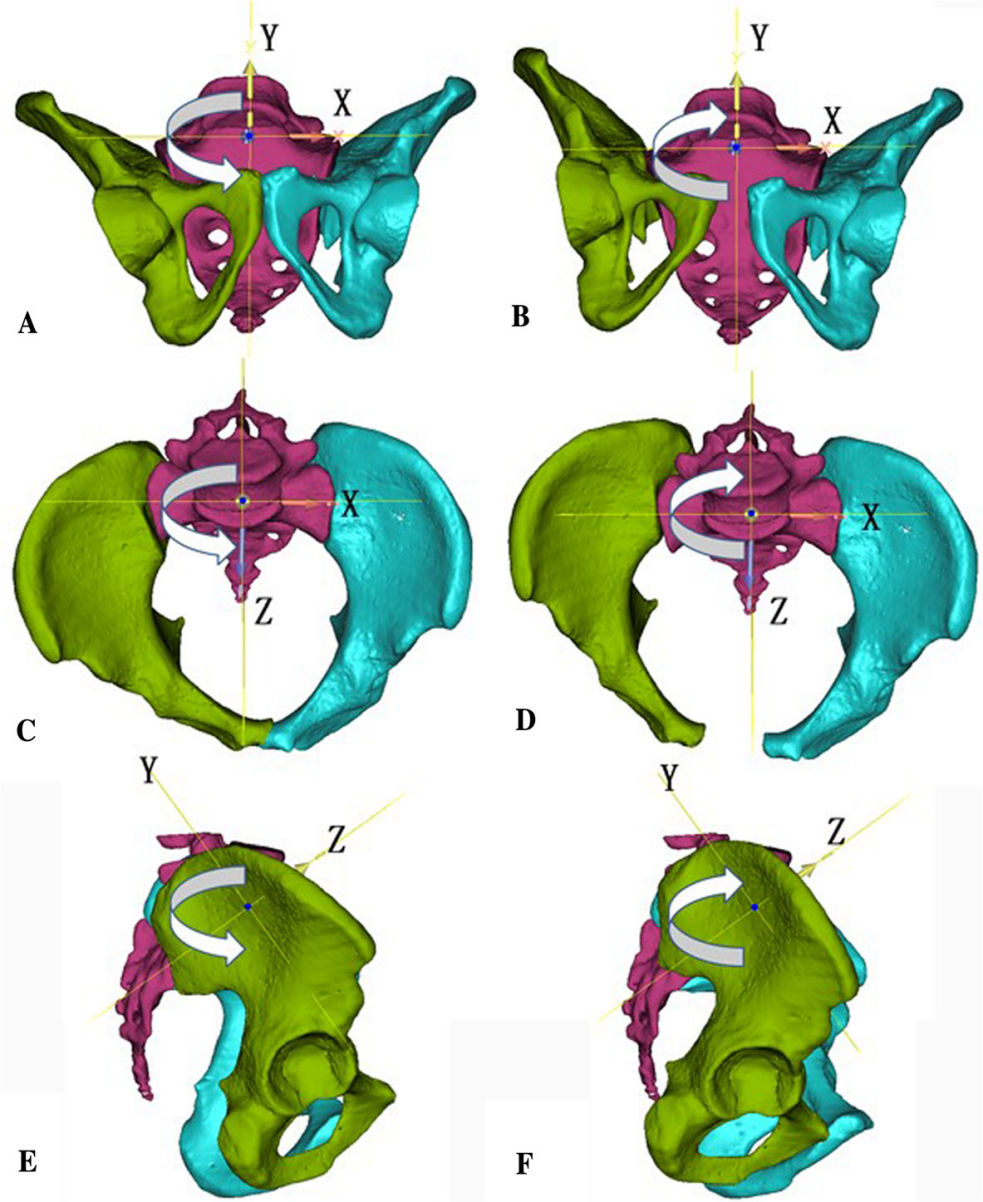
The rotational direction of the right hemipelvis is indicated by the curved arrow symbol in each pelvis, and the coordinate systems are denoted by the capital letters of X, Y and Z. **a** and **b** represent the varus and valgus of hemipelvis around the Z-axis in the outlet plane, respectively. **c** and **d** represent the internal and external rotation of the hemipelvis around the Y-axis in the inlet plane, respectively. **e** and **f** represent the flexion and extension of the hemipelvis around the X-axis in the sagittal plane, respectively

To better illustrate the rotational and translational displacement types, the pelvis specific coordination system was set up, in which the definition of all rotation types and the X/Y/Z coordinate system were defined (Fig. 1). In this system, the X/Z and X/Y planes represented the standard inlet and outlet radiographs of pelvis, respectively.

To present the relationship of the anatomic points between the intact and the injured hemipelvis, the standard radiographs have to be established at first. In this study, we imported the CT-DICOM data into Armira software (Visage Imaging GmbH, Berlin, Germany) to simulate the standard inlet, outlet, and AP radiographs of pelvises, which was defined as the standardized X-ray group.

### Representative anatomic landmarks

**Fig. 2:**
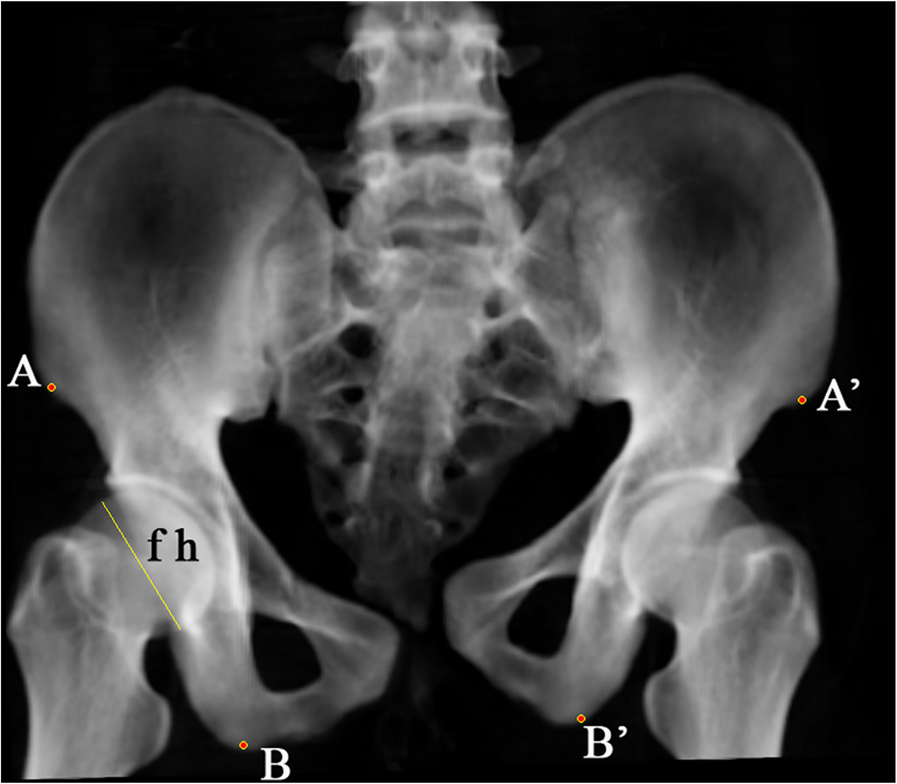
Anatomic landmarks in AP view: ASIS (A and A’), ischial tuberosity (B and B’). The right femoral head diameter is indicated by and was measured using line “fh”

**Fig. 3:**
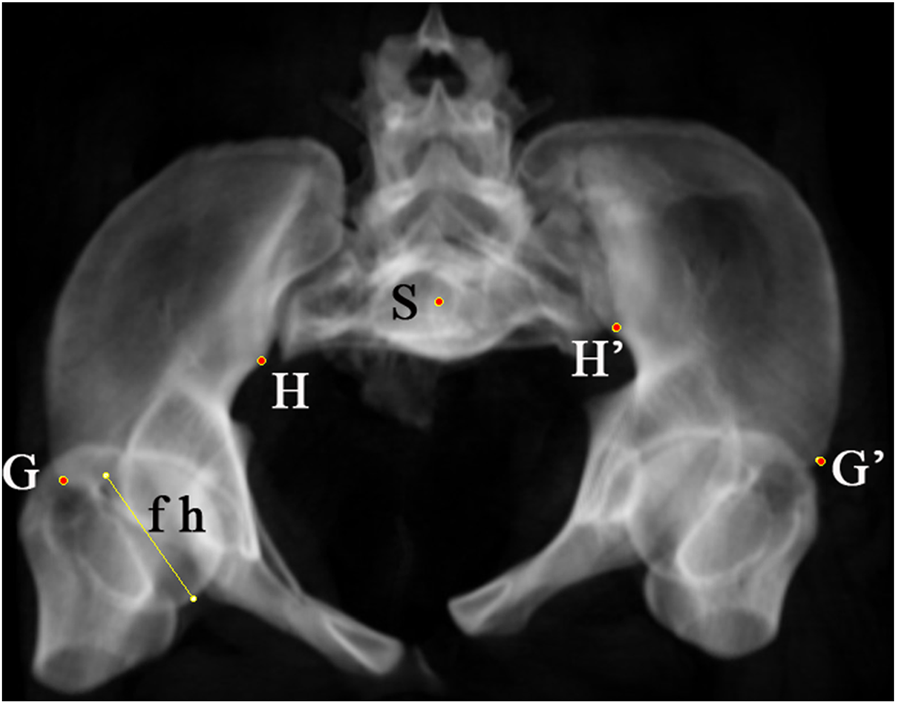
Anatomic landmarks in inlet view: ASIS (E and E’), superior point of iliac wing (C and C’), ischial tuberosity (D and D’) and centre of sacral endplate (S). The right femoral head diameter is indicated by and was measured using line “fh”

**Fig. 4:**
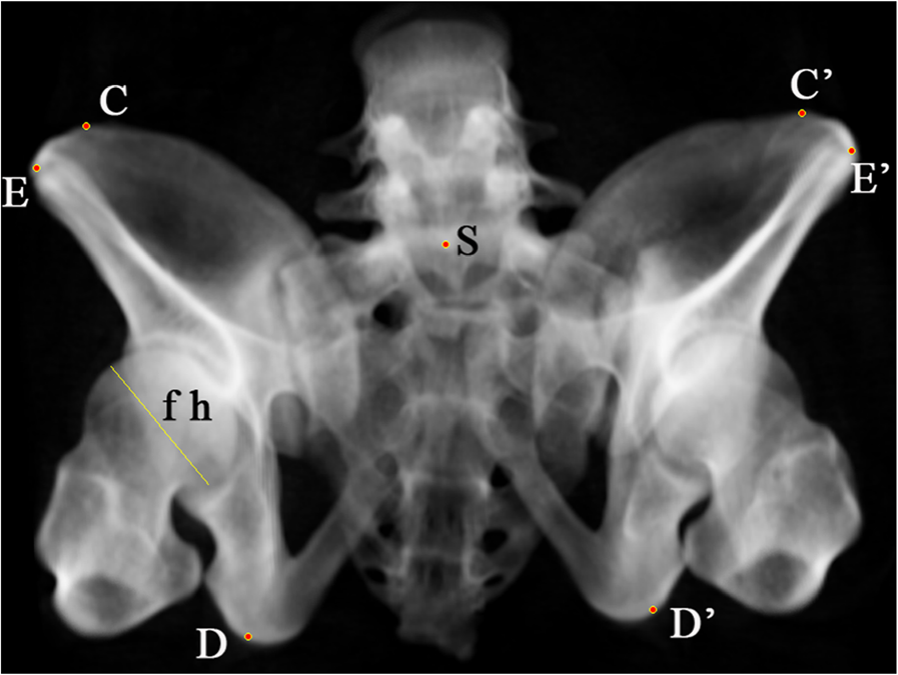
Anatomic landmarks in outlet view: ASIS (G and G’), anterior SI joint (iliac side) (H and H’), center of sacral endplate (S). The right femoral head diameter is indicated by and was measured using line “fh”

Several representative anatomic landmarks were selected using Digimizer 4.1.1.0 software (Model: Mitutoyo Corp., Tokyo, Japan) to measure and indicate the displacement type in inlet, outlet and AP images. In AP radiographs anterior superior iliac spine (ASIS) and ischial tubercle were marked to determine the rotation direction around the X-axis in the sagittal plane (Fig. 2). In inlet radiographs, the anterior SI joint (iliac side), ASIS and centre of sacral endplate were selected to determine the X-axis transverse and the Z axis AP displacement (Fig. 3). In outlet radiographs, the superior point of iliac wing was used to determine the vertical displacement, and ASIS and ischial tubercle were used to calculate the varus or valgus of the hemipelvis (Fig. 4).

### Standardization of the evaluation process

As the diameter of the intact femoral head in each case was different from the others, it was necessary to measure it using 3-matic (Materialise) in order to establish the relationship between pixel and actual distance, which could be used as the reference to set the size of the corresponding femoral head in each standardized radiograph. One independent investigator imported the CT scan data into Mimics 16 software (Materialise, Haasrode, Belgium) to create the 3D models of the fractured pelvises and measure the diameter of each femoral head, independently.

### The formulas for the translational and rotational displacement

The 2D coordinates of the previous mentioned anatomic landmarks in all three standard radiographs were acquired using Digimizer software and recorded in a Microsoft Excel file (Table 1). The following formulas were used as Excel commands and input into corresponding blanks, which could indicate the direction of displacement and calculate the actual parameters (Table 2). The translational displacement parameters of fractured hemipelvis were as follows.

**Table 1:** The representative anatomic landmarks used to measure the displacement of the hemipelvis in each projection

Radiographs	Anatomic landmarks	Intact side	Injured side
AP view	ASIS	X_A_,Y_A_	X’_A_,Y’_A_
	ischial tuberosity	X_B_,Y_B_	X’_B_,Y’_B_
Outlet view	superior point of iliac wing	X_C_,Y_C_	X’_C_,Y’_C_
	ischial tuberosity	X_D_,Y_D_	X’_D_,Y’_D_
	ASIS	X_E_,Y_E_	X’_E_,Y’_E_
	center of sacral endplate	X_S_,Y_S_	
Inlet view	ASIS	X_G_,Y_G_	X’_G_,Y’_G_
	anterior SI joint (iliac side)	X_H_,Y_H_	X’_H_,Y’_H_
	center of sacral endplate	X_S_,Y_S_	

AP anterior-posterior; ASIS anterior superior iliac spine

**Table 2:** Formulas to determine the displacement type

Type	Plane	Formulas and parameters	Direction	Related axes
Translational	Outlet	|Y’_C_-Y_S_|-|Y_C_-Y_S_| > 0	Cephlad	Y
		|Y’_C_-Y_S_|-|Y_C_-Y_S_| < 0	Caudad	Y
	Inlet	|Y’_H_-Y_S_|-|Y_H_-Y_S_| > 0	Anterior	Z
		|Y’_H_-Y_S_|-|Y_H_-Y_S_| < 0	Posterior	Z
		|X’_H_-X_S_|-|X_H_-X_S_| > 0	Lateral	X
		|X’_H_-X_S_|-|X_H_-X_S_| < 0	Medial	X
Rotational	Sagittal	|Y_A_-Y_B_|-|Y’_A_-Y’_B_| < −2	Flexion	X
		−2 ≤ |Y_A_-Y_B_|-|Y’_A_-Y’_B_| ≤ 2	Neutral	X
		|Y_A_-Y_B_|-|Y’_A_-Y’_B_| > 2	Extension	X
	Inlet	|X_G_-X_H_|-|X’_G_-X’_H_| < −2	External rotation	Y
		−2 ≤ |X_G_-X_H_|-|X’_G_-X’_H_| ≤ 2	Neutral	Y
		|X_G_-X_H_|-|X’_G_-X’_H_| > 2	Internal rotation	Y
	Outlet	|X_E_-X_D_|-|X’_E_-X’_D_| < −2	Valgus	Z
		−2 ≤ |X_E_-X_D_|-|X’_E_-X’_D_| ≤ 2	Neutral	Z
		|X_E_-X_D_|-|X’_E_-X’_D_| > 2	Varus	Z

Y-axis vertical displacement in outlet radiographs: |Y’_C_-Y_S_|-|Y_C_-Y_S_|.

Z-axis AP displacement in inlet radiographs: |Y’_H_-Y_S_|-|Y_H_-Y_S_|.

X-axis transverse displacement in inlet radiographs: |X’_H_-X_S_|-|X_H_-X_S_|.

The established inequalities to indicate the direction of rotation were based on the relative spatial positions between bilateral corresponding anatomic landmarks in 3D space during the movement of hemipelvis in a special direction (Table 2).

In the AP view, the vertical distance between ASIS and the ischial tubercle in the injured side smaller than that of the intact side could indicate extension of the injured hemipelvis in the sagittal plane, and vice versa. The difference between the vertical distances between ASIS and ischial tubercle in the injured side and that in the intact side could be used as an indicator of the sagittal rotational displacement of the injured hemipelvis (Fig. 2).

In the inlet view or the X/Z plane, the transverse distance between ASIS and the anterior SI joint (iliac side) in the injured side, smaller than that of the intact side, indicated internal rotation of the injured hemipelvis, and vice versa. The difference between the transverse distance between ASIS and the anterior SI joint (iliac side) in the injured side and that in the intact side could be used as an indicator of the rotational displacement of the injured hemipelvis in the inlet plane (Fig. 3).

In the outlet view or the X/Y plane, the transverse distance between ASIS and the ischial tubercle in the injured side smaller than that of the intact side indicated valgus of the injured hemipelvis, and vice versa. The difference between the transverse distance between ASIS and the ischial tubercle in the injured side and that in the intact side could be used as an indicator of the degree of varus and valgus of the injured hemipelvis in the outlet plane (Fig. 4).

### Reliability study

Five separate observers, including 2 full-time orthopaedic trauma surgeons (LHZ and XYS) and 3 orthopaedic trauma fellows (ZZ, LCZ and HLD), were recruited to perform this study.

Before actual measurements were performed, premeasurement assessment of the rotational direction in each plane using the 3D CT data was based on overall impression. Observers were asked to look through the AP views of all 3D CT reconstruction models of the pelvic fracture in a random order and an independent manner. It was stressed that this was their impression of the rotation direction, not based on actual measurement. They were asked not to change their answer after the measurements had been taken. After at least 3 days, the previously mentioned anatomic landmarks in all the standardized X-ray images in a random order were marked and determined the translational and rotational displacement using the established formulas by the same 5 doctors in an independent manner.

To compare the outcomes based on the standardized X-ray images, all 3D models of the fractured pelvises were reconstructed using Mimics software and the inlet, outlet, and AP views were simulated using 3-Matic software by one doctor (LNZ). The measurement outcomes were considered as the standard to test the standardized X-ray groups’ outcomes.

**Fig. 5:**
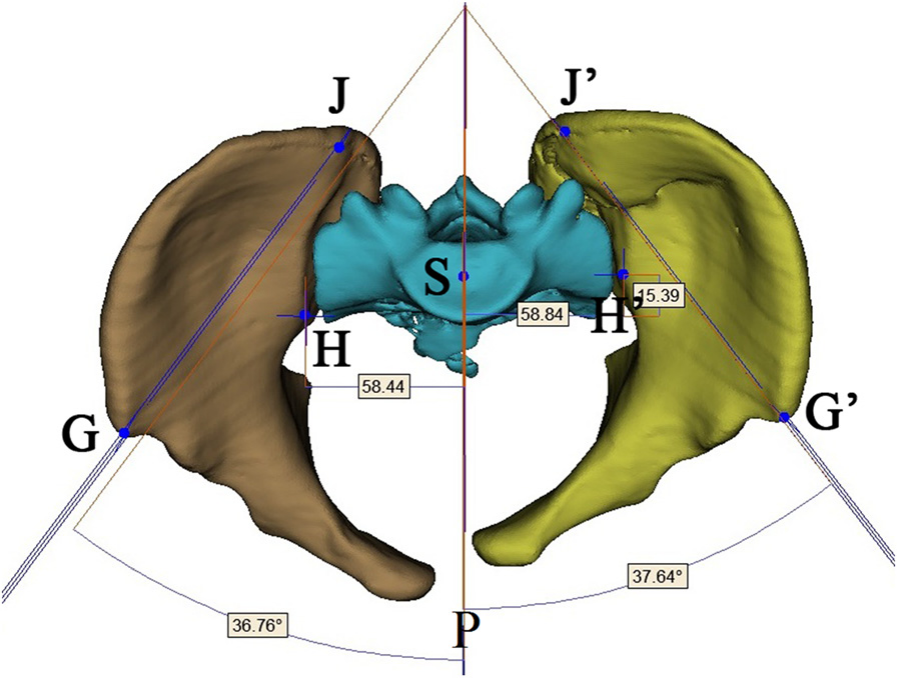
Anatomic landmarks in inlet view of 3D reconstruction model: anterior superior iliac spine (ASIS, G and G’), posterior superior iliac spine (PSIS, J and J’) anterior SI joint (iliac side) (H and H’) and centre of sacral endplate (S). The transverse and AP displacements and the degrees of internal and external rotation were measured using 3-matic measurement tool. The capital P represents the sagittal plane

**Fig. 6:**
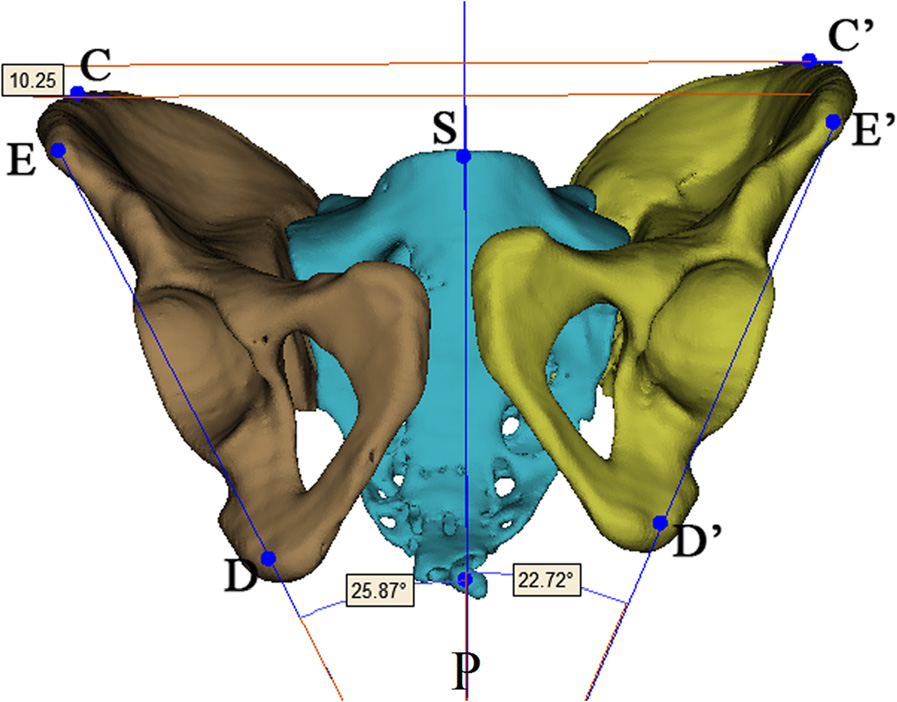
Anatomic landmarks in outlet view of 3D reconstruction model: ASIS (E and E’), centre of sacral endplate (S), ischial tuberosity (D and D’), and the software measurement method of vertical translational displacement and the degree of varus and valgus. The capital P represents the sagittal plane

**Fig. 7:**
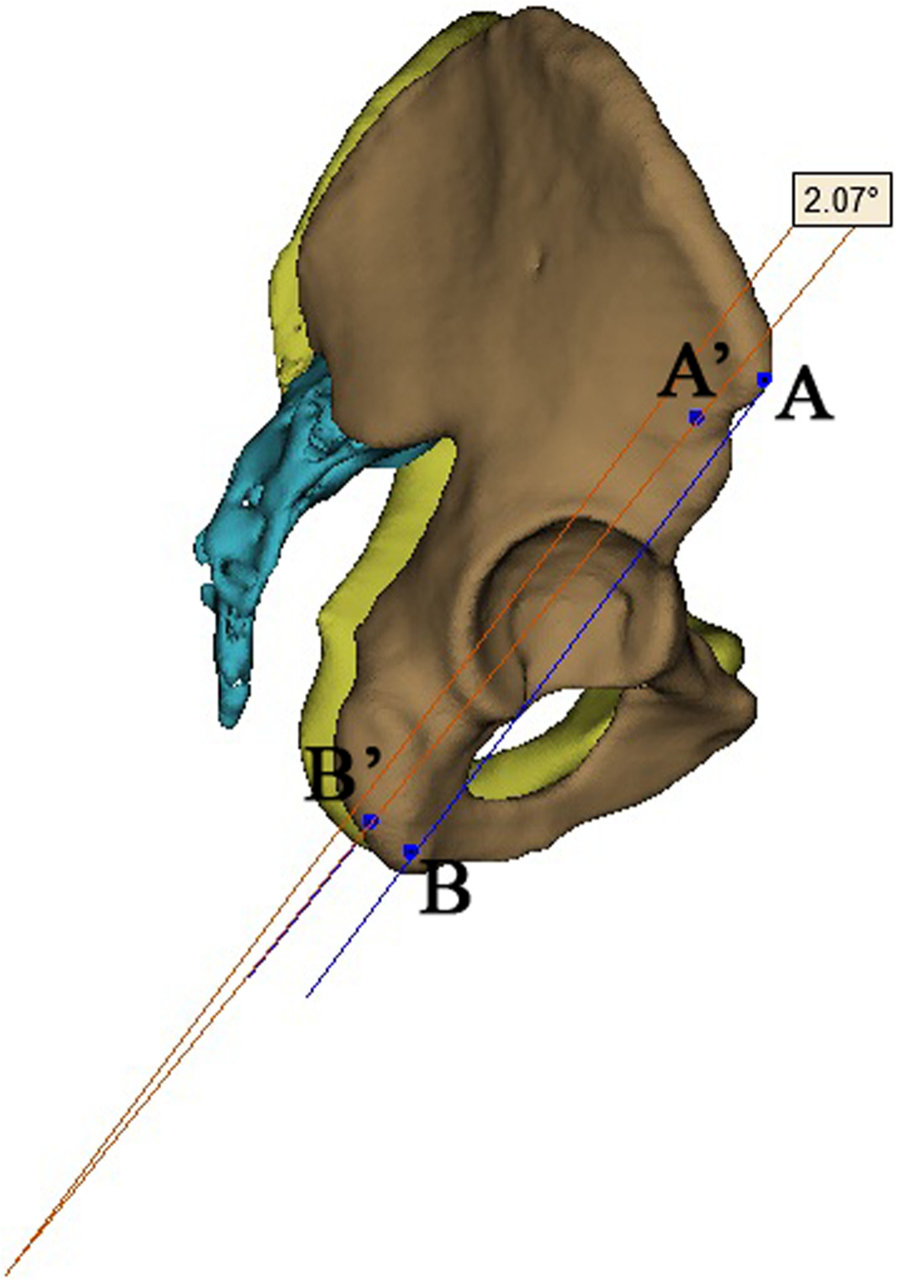
Anatomic landmarks in lateral view of 3D reconstruction model: ASIS (A and A’), ischial tuberosity (B and B’), and the measurement method of the degree of flexion or extension rotation using software tool

**Fig. 8:**
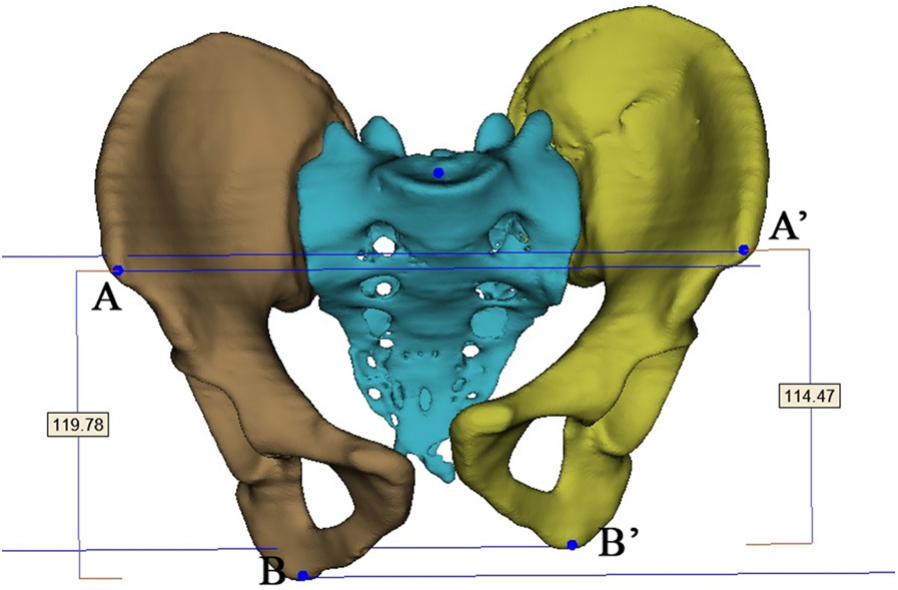
Anatomic landmarks in AP view of 3D reconstruction model: ASIS (A and A’), ischial tuberosity (B and B’), and the measurement results of parameters of flexion and extension rotation using 3-matic software

One orthopaedic surgeon (JXZ) simulated the inlet, outlet, and AP view and completed the calculation, independently. The methods of calculating the translational and rotational displacement in the outlet plane and the translational displacement in the inlet plane were the same as that used in the radiographic measurement (Figs. 5 and 6). In the inlet plane, posterior superior iliac spine was introduced and marked with the ASIS to indicate the internal or external rotational displacement in the same calculation method. (Figs. 5) In the sagittal plane, the flexion and extension around the X-axis were indicated by the relationship between bilateral lines extending from the respective iliac superior point and ischial tubercle (Fig. 7). The 2D indicator of the sagittal rotational displacement was also calculated in the AP view (Fig. 8).

### Statistical analysis

Agreement between observers for categorical and continuous outcomes was calculated using kappa statistic [12] and intraclass correlation coefficient (ICC) by Minitab 16 (Minitab Inc., State College, PA, USA) and SPSS 20 software (SPSS Inc., Chicago, IL, USA), respectively. According to Shrout et al. [13], the two-way random model of ICC was selected to assess the interobserver reliability. In both instances, the strength of agreement was determined based on a standardized Landis-Koch scale (0, poor; 0 to 0.2, slight; 0.21 to 0.40, fair; 0.41 to 0.60, moderate; 0.61 to 0.80, substantial; 0.81 to 1.0, almost perfect) [14].

## Results

The 25 patients included in this study were of a mean age of 37.4 years at the time of injury, with 17 men and 8 women. The case series included AO/OTA type C (vertical shear) and type B (AP or lateral compression) with unilateral pelvis injured and dislocated.

The evaluation of the translational measurement outcome of the standardized X-rays showed an overall very good agreement between observers with an ICC of 0.9 and 95 % confidential interval greater than 0.75, as seen in Table 3. Table 4 showed that the agreement of rotational measurement outcomes of the standardized X-rays with an ICC of 0.9 (almost perfect). The interobserver reliability of the reader’s impression of the rotational results based on the 3D-CT model was fair overall with a kappa statistic between 0.265 and 0.426 (Table 5), among which the agreement of full-time orthopaedic trauma surgeons was higher than that of orthopaedic trauma fellows, with average kappa statistics of 0.428 and 0.162, respectively (not shown in table).

**Table 3:** ICC for translational displacement in the standardized X-ray

Displacement	ICC Coefficient	95 % CI
Vertical	0.920	0.856–0.963
AP	0.934	0.878–0.974
Transverse	0.945	0.893–0.972

AP anterior-posterior; ICC intraclass correlation coefficient

**Table 4:** ICC for rotational displacement in different planes in the standardized X-ray

Displacement	ICC Coefficient	95 % CI
Sagittal	0.947	0.872–0.997
Inlet	0.925	0.887–0.976
Outlet	0.903	0.861–0.957

ICC intraclass correlation coefficient

**Table 5:** Agreement for rotational displacement based on the first impression of the AP view of 3D CT images

Displacement	Kappa	*P* value
Sagittal	0.250	<0.001
Inlet	0.426	<0.001
Outlet	0.265	<0.001

As the Majeed scale defines 0–2 mm displacement as the anatomic reduction of a fractured hemipelvis [15], we defined the absolute value of rotational parameter between 0 and 2 calculated in 3D model by 3-matic as neutral position. Based on this precondition, the assessment of the rotational parameters between the impression outcome of the 3D model and the displacement measurement calculated by 3-matic showed that the interobserver reliability was in a wide range between 0.634 and 0.169 (Table 6). It can be seen that the agreement of full-time orthopaedic trauma surgeons (number 1–3) was higher than that of orthopaedic trauma fellows (number 4, 5) (Table 6).

**Table 6:** Agreement for rotational displacement between the first impression of the 3D CT images and the measurements of the 3D reconstruction models

Doctor	Displacement plane	Kappa	*P* value
1	Sagittal	0.269	0.030
	Inlet	0.202	0.088
	Outlet	0.307	0.022
2	Sagittal	0.384	<0.001
	Inlet	0.540	<0.001
	Outlet	0.340	0.008
3	Sagittal	0.211	0.069
	Inlet	0.169	0.125
	Outlet	0.196	0.104
4	Sagittal	0.634	<0.001
	Inlet	0.493	<0.001
	Outlet	0.438	0.001
5	Sagittal	0.451	<0.001
	Inlet	0.549	<0.001
	Outlet	0.454	<0.001

As the rotational direction assessed in the standardized X-rays was the same as that of 3D reconstruction group, the interobserver agreement between both groups was evaluated using rotational displacement parameters calculated by formula. Table 7 and 8 showed the interobserver agreement of rotational and translational displacement parameters with both the ICCs being higher than 0.9 (almost perfect).

**Table 7:** Agreement for rotational displacement between the standardized X-ray and 3D reconstruction

Doctor	Displacement plane	ICC	95 % CI
1	Sagittal	0.929	0.877–0.964
	Inlet	0.913	0.891–0.995
	Outlet	0.947	0.896–0.981
2	Sagittal	0.911	0.843–0.945
	Inlet	0.903	0.865–0.957
	Outlet	0.939	0.867–0.978
3	Sagittal	0.977	0.941–0.997
	Inlet	0.935	0.899–0.971
	Outlet	0.933	0.871–0.973
4	Sagittal	0.929	0.874–0.969
	Inlet	0.957	0.902–0.995
	Outlet	0.907	0.885–0.959
5	Sagittal	0.918	0.871–0.961
	Inlet	0.971	0.931–0.996
	Outlet	0.951	0.895–0.979

ICC intraclass correlation coefficient

**Table 8:** ICC for translational displacement between the standardized X-ray and 3D reconstruction

Doctor	Displacement direction	ICC Coefficient	95 % CI
1	Vertical	0.994	0.984–0.998
	AP	0.943	0.898–0.974
	Transverse	0.977	0.967–0.998
2	Vertical	0.987	0.958–0.994
	AP	0.957	0.935–0.984
	Transverse	0.961	0.931–0.985
3	Vertical	0.953	0.936–0.989
	AP	0.945	0.907–0.977
	Transverse	0.911	0.887–0.953
4	Vertical	0.932	0.897–0.976
	AP	0.935	0.895–0.965
	Transverse	0.959	0.941–0.989
5	Vertical	0.928	0.887–0.978
	AP	0.974	0.947–0.996
	Transverse	0.985	0.975–0.992

AP anterior-posterior; ICC intraclass correlation coefficient

## Discussion

Currently, radiographic assessment remains the standard for preoperative assessment and the most frequently used outcome measure during studies of the pelvis. As with other outcome measurement tools, reproducibility, reliability and validity of radiographic measurement techniques are of utmost importance if there is an intention to use these to assess outcome. However, many authors argue that the current measurement methods for fractured pelvises lack standardization, well-accepted reliability, and validity [8, 10].

A literature review by Mataliotakis et al. on the radiographic measurement of pelvic fractures showed various measurement systems introduced by different authors [16]. Radiological evaluation on vertical pelvic displacement was mainly dependent on AP view of the pelvis [3, 7–9]. Matta et al. and Dickson et al. measured the vertical displacement as the difference in the height of the femoral heads in AP view [4, 7]. Henderson et al. and Griffin et al. also used the AP view to measure the perpendicular difference between the most superior points of the bilateral iliac wings as the vertical displacement [3, 17]. Sagi et al. modified the method of Matta and Tornetta and used the superior edge of the femoral head in the outlet radiograph to determine the vertical displacement of the displaced hemipelvis [18]. Lefaivre et al. used the top of the iliac crests in the outlet film to determine the vertical displacement of the displaced hemipelvis relative to its counterpart [8].

Almost all methods for the vertical displacement measurement used by authors were dependent on the pelvic AP X-ray. Actually, both the AP and the outlet radiographs could be used to measure the vertical displacement of the pelvis. The main difference is that which reference system is selected beforehand. With regard to the movements in 3D space, the best method to depict and determine it is to establish a Cartesian coordinate system which includes three mutually orthogonal axes, such as the X, Y and Z axes. If the vertical displacement of the pelvis is measured on the AP radiograph, it means that the vertical axis is considered as an axis of the rotational or translational movements, and another two axes have to be determined using the transverse and the body’s anterior-posterior axes. However, the types of open and close book of the pelvic injuries are the most frequently mentioned type of pelvic injuries which compose almost all the Young-Burgess lateral compression type of pelvic injuries. The open and close book of the pelvic injuries are mainly determined and better demonstrated on the inlet radiographs, which could be considered as the external or internal rotation of the displaced hemipelvis around the axis across the centre of the sacrum and perpendicular to the inlet plane. Based on this precondition, another two axes could be established perpendicular to this axis at the same time, which are the body’s transverse axis and the axis perpendicular to the outlet plane.

Another reason why the physiologic coordination is not chosen is that the oblique degrees of the pelvis are inconsistent among people and vary with a range between 40° and 60° [19], but the pelvis specific coordination could be considered as a relative objective reference system to depict and measure the movement type of the pelvis itself. Thus, although almost all methods for the vertical displacement measurement used by authors were dependent on the pelvic AP X-ray, the measurement results based on the AP view might not correspond to the true one. Boontanapibul et al. compared hemipelvic vertical displacement measurements results based on the pelvic outlet and AP radiographs, and concluded that pelvic outlet radiograph could provide efficient measurements of hemipelvic vertical displacement, but the measurements results based on the AP radiographs were inconsistent [20].

In addition, as severe complications and multiple injuries are common in high-energy pelvic fractures, compared to the hip joint, which is easy to implicate and fracture, the iliac crest seems to be a more consistent anatomic landmark to use as the reference to measure the displacement. Consequently, it is more reliable and precise to reflect the vertical displacement of hemipelvis by measuring the positions of bilateral iliac crests’ superior points in outlet view rather than measuring the hip joints’ positions in the AP view.

For the measurement of AP displacement of pelvis, Henderson et al. used both ischial spine positions in the inlet view as the bony landmark [3]. Sagi et al. also used the inlet radiograph for the AP displacement [18]. Lindahl et al. used pelvic CT scans’ axial sections for the measurement of the AP displacement, even though no details of measurement methods were available [21].

Regarding the rotational displacement, current inconsistent evaluation methods mainly focused on the rotation in the unilateral plane. Lefaivre et al. evaluated the rotational displacement of the hemipelvis in the horizontal plane by measuring the pubic symphysis diastasis in AP X-rays. Dickson et al. measured the difference between the width of bilateral ischiums as the reference reflecting the rotational direction of hemipelvis, and measured the angle between the midline and the quadrilateral plate of the injured side in the CT scan axial section to represent the rotational angle of the hemipelvis [4]. Sagi et al. measured the perpendicular distances between the acetabulum and sacrum in inlet and outlet views, separately, and evaluated the asymmetry of the pelvis in inlet and outlet views by calculating the ratio between the bilateral distances in the respective plane, which was also named as the inlet/outlet ratio method [18]. Keshishyan et al. evaluated the pelvic asymmetry by calculating the ratio between the distances of the inferior aspect of the sacroiliac joint to the inferior aspect of the contralateral teardrop on both sides of the pelvis. As the pelvis represents a three-dimensional structure, any rotational measurements applied should be as representative as possible to its three dimensions. Although the present different methods for rotation displacement were complicated, there has not been a well-accepted validated method thus far. Nystrom et al. developed a rotational assessment method based on the computer-reconstructed radiographs [1]. However, in their study, the pelvic rotational displacements were only measured around two axes perpendicular to the inlet and sagittal planes, respectively.

Lefaivre et al. analyzed almost all the radiographic measurement methods for the rotational displacement of the pelvic fracture and concluded three most reliable methods [22], including the Sagi, the Keshishyan or Smith and the Lefaivre methods [8, 18, 23, 24]. Hereafter, they performed a comparative study to test the interobserver reliability of three commonly used radiographic measurement methods [10]. The cross measurement technique of Keshishyan showed that overall there is very good agreement between observers, with an ICC coefficient above 0.9 [24]. The inlet and outlet ratio method of Sagi showed less interobserver reliability with a coefficient between 0.5 and 0.8 [18]. The absolute displacement methods described by Lefaivre et al. showed the poorest interobserver agreement [8]. The authors analyzed and noted that the more reference lines or marks and the more steps used, the poorer the agreement would be concluded and the greater the error would be magnified.

The aim of this study was to set up a measurement system, which could allow observers to complete the measurement of displacement in a more convenient manner within as few steps as possible. Ideally, it could minimize the disagreement and errors brought by multiple steps and precisely indicate the rotational direction in different planes, which could be used as the basis for preoperative assessment and treatment plan.

As unstable pelvic fractures are classified as a pelvis with the complete disruption of the posterior osseous ligamentous structure, the posterior pelvic ring was the keystone for preoperative assessment and treatment plans. Thus, the measurement technique of the pelvic translational displacement should focus on the area around the SI joint or posterior pelvic ring. Furthermore, we studied the relationship between the positions of bilateral corresponding anatomic landmarks in 3D coordinate systems and the motion of the hemipelvis in different directions, set up a series of formulas to indicate the rotational displacement around three mutually perpendicular axes, and calculated the associated displacement parameters. Regarding the three-dimensional structure of pelvis, to minimize the measurement error, we selected three landmarks with different 3D coordinates dispersed far away from each other, such as ASIS, the iliac aspect of the SI joint, and ischial tubercle. By comparing with the coordinates of the corresponding anatomic landmarks in bilateral hemipelvis, we concluded a series of formulas that could indicate the rotational direction of the injured hemipelvis, based on the variation of the difference between the distance between landmarks of the injured side in one plane and the distance between the same corresponding landmarks of the intact side in the same plane, following the rotation of injured hemipelvis in the same specified plane. For example, if the injured hemipelvis flexed in the sagittal plane, the distance between the ASIS and ischial tubercle would be reduced compared with the distance between corresponding points on the opposite side, and vice versa. The same situation happened in other types of motions in the corresponding plane.

After the previous step where the formula is established, we also need the ratio between the actual distance and the measurement on the X-ray images, by acquiring and setting each femoral head diameter through measuring its 3D model using 3-matic software, and the relationship between the measurement on the X-ray images and the distance between pixels on the 2D coordination system. In the end, the process of all relevant cumbersome calculations could be simplified by the acquisition of special anatomic landmarks’ 2D coordinates in the corresponding planes and carried out in a few mouse clicks. Ideally, this method could reflect the actual spatial rotational displacements of injured hemipelvis more precisely, and direct the manual reduction manipulations in the clinical practices. However, as was the case in most previous measurement methods, the premise of the current measurement system is that the injured hemipelvis was relatively intact. The proposed measurement method could be used by all the researchers, medical students, radiologists and orthopedic surgeons who are familiar with the pelvic anatomic morphology and landmarks and the injury mechanisms, including AO/OTA and Young-Burgess classifications, and displacements characteristics of the unstable pelvic fractures.

Viegas et al. demonstrated that the three-dimensional CT scan reconstruction technique could provide the bone measurement a volumetric accuracy of 94 % and a linear accuracy of 97 % [25]. Nystrom et al. also tested and verified the validity of CT based measurements of fracture displacement [11]. Thus, the measurement on the 3D CT model was regarded as the standard to test the measurements on the standardized X-ray images. According to the results, interobserver reliability of either translational or rotational measurement was high as both ICCs were higher than 0.9, which means the agreement was nearly perfect and would allow subsequent authors to repeat it.

## Conclusion

The new method for measuring translational and rotational displacement based on the established formula is reliable and can minimize the errors in pelvic fracture measurements. Furthermore, this study was possible for the standardization of the preoperative plan and used as the theoretical basis for initiating a process of robot-assisted pelvic fracture surgery based on two-dimensional radiographs.

## Abbreviations

AP: Anterior-posterior
2D: 2-dimensional
3D: 3-dimensional
ICC: Intraclass correlation coefficient
SI: Sacroiliac
DICOM: Digital-imaging-and-communications-in-medicine
ASIS: Anterior superior iliac spine
